# Evaluation of two sexual-stage antigens as bivalent transmission-blocking vaccines in rodent malaria

**DOI:** 10.1186/s13071-021-04743-0

**Published:** 2021-05-07

**Authors:** Fan Yang, Fei Liu, Xinxin Yu, Wenqi Zheng, Yudi Wu, Yue Qiu, Ying Jin, Liwang Cui, Yaming Cao

**Affiliations:** 1 Department of Immunology, College of Basic Medical Sciences, China Medical University, Shenyang, 110122 Liaoning China; 2 Department of Laboratory Medicine, The Affiliated Hospital of Inner Mongolia Medical University, Hohhot, China; 3 The First Affiliated Hospital of China Medical University, 155 Nanjing North Street, Heping District, Shenyang, 110001 Liaoning China; 4 Liaoning Research Institute of Family Planning, Shenyang, 110031 China; 5 Department of Internal Medicine, Morsani College of Medicine, University of South Florida, Tampa, FL 33612 USA

**Keywords:** Transmission-blocking vaccine, Dual-antigen, Immunological interference, Transmission-blocking activity

## Abstract

**Background:**

Transmission-blocking vaccine (TBV) is a promising strategy for malaria elimination. It is hypothesized that mixing or fusing two antigens targeting different stages of sexual development may provide higher transmission-blocking activity than these antigens used individually.

**Methods:**

A chimeric protein composed of fragments of Pbg37 and PSOP25 was designed and expressed the recombinant protein in *Escherichia coli* Rosetta-gami B (DE3). After immunizing mice with individual recombinant proteins Pbg37 and PSOP25, mixed proteins (Pbg37+PSOP25), or the fusion protein (Pbg37-PSOP25), the antibody titers of individual sera were analyzed by ELISA. IFA and Western blot were performed to test the reactivity of the antisera with the native proteins in the parasite. The transmission-blocking activity of the different immunization schemes was assessed using in vitro and in vivo assays.

**Results:**

When Pbg37 and PSOP25 were co-administered in a mixture or as a fusion protein, they elicited similar antibody responses in mice as single antigens without causing immunological interference with each other. Antibodies against the mixed or fused antigens recognized the target proteins in the gametocyte, gamete, zygote, and ookinete stages. The mixed proteins or the fusion protein induced antibodies with significantly stronger transmission-reducing activities in vitro and in vivo than individual antigens.

**Conclusions:**

There was no immunological interference between Pbg37 and PSOP25. The bivalent vaccines, which expand the portion of the sexual development during which the transmission-blocking antibodies act, produced significantly stronger transmission-reducing activities than single antigens. Altogether, these data provide the theoretical basis for the development of combination TBVs targeting different sexual stages.

**Graphic Abstract:**

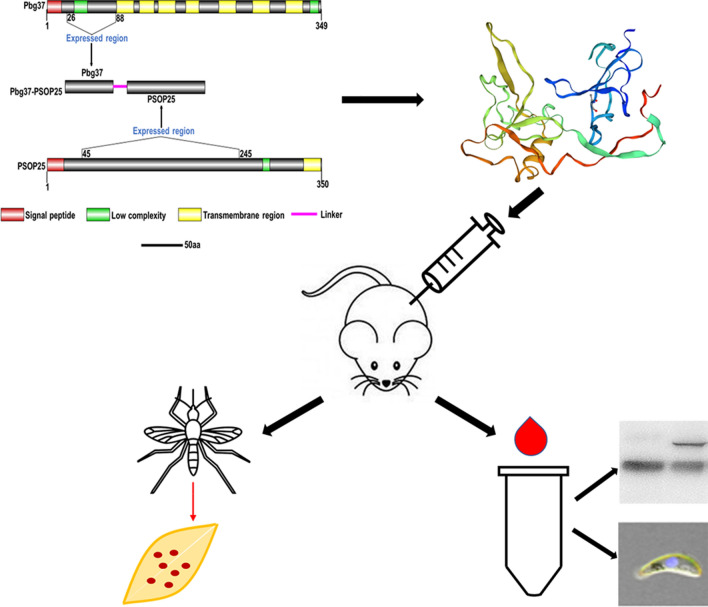

**Supplementary Information:**

The online version contains supplementary material available at 10.1186/s13071-021-04743-0.

## Background

Malaria is one of the most serious infectious diseases impacting global public health and economic development. According to the World Malaria Report 2020, there were 229 million malaria cases and 409,000 malaria deaths globally in 2019 [1]. Malaria control measures such as insecticide-treated bed nets, indoor residual sprays of insecticides, and artemisinin combination therapies have collectively contributed to a significant decrease in the morbidity and mortality from malaria. However, the emergence of drug-resistant parasites and insecticide-resistant mosquitoes poses great challenges to malaria control and elimination [1]. Vaccines, in general, have been a highly successful intervention, but mainly against many viral diseases. By contrast, efforts to develop an effective vaccine against malaria infection or transmission have not yet succeeded [2]. Among the vaccine designs against malaria parasites, transmission-blocking vaccines (TBVs), which target the sexual and/or sporogonic development of the parasite, are intended to reduce the transmission of malaria parasites from humans to mosquitoes [3].

The malaria parasite has a complex life cycle, including developmental stages in both the human host and the mosquito vector. The transmission of malaria begins with the formation of the sexual precursor stage, gametocytes, in humans. Once ingested by a mosquito, male and female gametocytes, experiencing environmental changes such as a lower temperature, higher PH, and the presence of xanthurenic acid, are activated to form gametes, which fertilize to form a diploid zygote inside the midgut. Within 24 h, the zygote transforms into a motile ookinete, which penetrates the midgut epithelium to develop into an oocyst under the basal lamina [4]. Over the next 2 weeks, each oocyst produces thousands of sporozoites, which migrate to the salivary glands and become ready to be transmitted during subsequent bites of the mosquito [5, 6]. Antigens expressed during the sexual development of the malaria parasites, either expressed in gametocytes or gametes, are called pre-fertilization antigens, while those expressed in zygotes and ookinetes are considered post-fertilization antigens [7].

The fundamental principle of TBVs is to immunize humans with sexual-stage surface antigens of the parasites to produce antibodies that arrest subsequent development of the parasites in mosquitoes. Although TBVs do not directly protect vaccinated people from the morbidity of malaria, they play a key role in controlling the spread of the parasites in a community [8]. Several promising candidates have been investigated for TBV development, including the pre-fertilization antigens P230, P48/45, and HAP2, and the post-fertilization antigens P25 and P28. P48/45 and P230 are essential for the adhesion of male gametes to female gametes. Antibodies against pre-fertilization antigens such as P48/45 are found in human sera from endemic areas and correlate with transmission-blocking activity (TBA) [9, 10]. Immunization against the first cysteine-motif domain of Pfs230 and the conserved HAP2 *cd* loop peptides can elicit antibodies with strong TBA [11, 12]. The post-fertilization antigens P25 and P28 have received much attention, and immunization against recombinant P25 and P28 can completely inhibit parasite development in mosquitoes [13]. To date, Pfs25 and Pvs25 have been studied in several clinical trials [14, 15]. However, most of the TBV candidates could only induce incomplete blocking of malaria transmission [16]. Thus, efforts have been undertaken to discover additional antigens and develop immunization methods to enhance antibody production [17].

Since subunit vaccines based on a single malaria antigen may fail to produce 100% efficacy, a multi-antigen and multi-stage vaccine, in which immune responses are elicited against more than one antigen and antigens from different stages of the parasite life cycle, might be a more effective vaccination strategy. Several studies have investigated whether a multiple antigen combination would be able to enhance the immune efficacy beyond that of single antigens or whether the inclusion of multiple antigens could cause immune interference [18]. It has been shown that the combination of two blood-stage antigens MSP1 and AMA1 caused immune interference by the immunodominant antigen [19], whereas the two ookinete antigens Pfs25 and Pfs28 did not show immune interference [20–22]. Furthermore, two studies showed that dual-antigen vaccines based on Pfs25 and Pfs230C did not elicit better transmission-reducing activity (TRA) than the mono-antigen vaccines [18, 23]. However, the Pfs230 and Pfs48/45 fusion proteins were found to elicit functional antibodies in mice with higher TBA than the single proteins alone [24]. These studies suggest that the strength of functional TBA from vaccination with multiple antigens may depend on the specific antigens used in the combination and how they are combined.

Several new TBV candidates were identified recently, including a gametocyte plasma membrane protein Pbg37 and an ookinete surface protein PSOP25 [25, 26]. The recombinant Pbg37 protein targeting the N-terminal 63 amino acids (aa) was able to elicit strong antibody responses with TBA. Consistent with it being a pre-fertilization antigen, the major inhibitory effects of the Pbg37 antisera were on the exflagellation and fertilization processes [25]. Similarly, antisera against the aa 45–245 fragment of PSOP25 also showed significant in vitro and in vivo TBA [26]. Here, this study aims to evaluate whether the combination of these two new antigens targeting different stages of sexual development could improve TBA.

## Methods

### Experimental mice, parasite line, and mosquitoes

Female, 6–8-week-old BALB/c mice were purchased from the Beijing Animal Institute (Beijing, China). The *Plasmodium berghei* ANKA 2.34 strain was maintained by serial passages. *Anopheles stephensi* mosquitoes (Hor strain) were bred for a 12 h light–dark cycle at 50–80% humidity and 25 °C. All experiments with animals were performed following the rules of the Animal Ethics Committee at China Medical University.

### Expression and purification of recombinant protein

To generate a chimeric Pbg37-PSOP25 protein, the gene fragments of Pbg37 (aa 26–88) and PSOP25 (aa 45–245) were fused with a flexible linker (GGGGS)_3_ between the two sequences by overlapping polymerase chain reaction (PCR) using primers listed in Additional file 1: Table S1. The PCR products were cloned into the vector *pET32a* (+) (Novagen, USA). Recombinant proteins were all expressed in *Escherichia coli* Rosetta-gami B (DE3) cells under the induction with 1 mM isopropyl β-d-1-thiogalactopyranoside (Sigma, USA) and 1% glucose at 19 °C for 8 h. Individual proteins of Pbg37 (~7 kDa) and PSOP25 (~22 kDa) as well as the Pbg37-PSOP25 chimera were generated as fusion proteins with thioredoxin (Trx, ~20 kDa) [25–27]. The His-tagged Trx was also expressed and used as the immunization control [25]. The bacteria were harvested by centrifugation and resuspended in a binding buffer containing 10 mM imidazole, 50 mM sodium phosphate buffer, and 300 mM NaCl (pH 8.0). The suspension was sonicated for 15 cycles (20 s pulses with 30 s intervals between each cycle). The lysates were passed through a 0.22 μm filter and incubated with Ni–NTA His-Bind Superflow resin (Novagen, USA) at 4 °C for 1 h. The resin was washed three times with the washing buffer containing 20 mM imidazole, 50 mM sodium phosphate buffer, and 300 mM NaCl (pH 8.0) and then eluted with an elution buffer containing 250 mM imidazole, 300 mM NaCl, and 50 mM sodium phosphate buffer (pH 8.0). The eluent containing purified protein was desalted by dialyzing extensively in 0.1 M phosphate-buffered saline (PBS) at 4 °C overnight. The recombinant fusion proteins were examined on a 10% sodium dodecyl sulfate-polyacrylamide gel electrophoresis (SDS-PAGE) gel under reducing conditions [26]. For enzyme-linked immunosorbent assay (ELISA) of antiserum titers, the recombinant Pbg37 (~7 kDa) and PSOP25 (~22 kDa) were digested with enterokinase (Solarbio, China) to remove the Trx tag.

### Immunization procedure

BALB/c mice (10 per group) were subcutaneously immunized with 50 μg of Pbg37, 50 μg of PSOP25, mixed (50 μg Pbg37+50 μg PSOP25), or 50 μg chimeric (Pbg37-PSOP25) recombinant proteins, emulsified with complete Freund’s adjuvant. Subsequently, the mice were immunized by two boosters at 2-week intervals with 25 μg of Pbg37, 25 μg of PSOP25, mixed (25 μg Pbg37+25 μg PSOP25), or 25 μg Pbg37-PSOP25 chimeric recombinant proteins, emulsified with incomplete Freund’s adjuvant. For antisera, blood was collected 10 days after the third immunization and allowed to clot at room temperature [23, 28]. Immunization with the recombinant Trx-His was used as a control.

### ELISA

The antibody titers were analyzed by ELISA as in a previous study [26]. The 96-well plates were coated with 10 μg/mL of purified Pbg37 or PSOP25 recombinant proteins after the removal of the Trx tag in 0.05 M sodium carbonate buffer at 4 °C overnight and then washed twice by 200 μL washing buffer (0.1 M PBS with 0.02% Tween 20, pH 7.4). The plates were blocked for 1 h at 37 °C with 1% bovine serum albumin (BSA, Sigma) dissolved in PBS. After three washes with the washing buffer, 100 μL individual mouse serum or mixed sera of ten mice per group, serially diluted with 1% BSA/PBS from 1:1000 to 1:128,000, was added into each well of the plate and incubated for 2 h at 37 °C. After three washes, 100 μL horseradish peroxidase (HRP)-conjugated goat anti-mouse IgG antibodies (1:5000 dilution, Invitrogen, USA) was added and incubated for 2 h at 37 °C. Then the plates were washed five times, and 100 μL of tetramethylbenzidine was added (Amresco, USA) and incubated for 10 min. Finally, 50 μL of 2 N H_2_SO_4_ was added to terminate the reaction, and each plate was read at 450 nm using a microplate reader. The value of the antibody titer was defined as the final dilution of a serum sample at which it had an optical density (OD) value no less than the average value of control antisera + 3 standard deviations [25].

### Western blot

To confirm the expression of Pbg37-PSOP25, 2 μg of the purified recombinant proteins was electrophoresed on 10% SDS-PAGE gels under reducing conditions and then transferred to a PVDF membrane (Bio-Rad, USA). Membranes were blocked with 5% nonfat milk in Tris-buffered saline with 0.1% Tween 20 (TBST) at room temperature for 2 h, and then incubated with an anti-His monoclonal antibody (1:2000, ABclonal, China) for 2 h. To determine whether the antibodies recognized the *P. berghei* native proteins, gametocytes and ookinetes were purified [29], and parasite lysates were prepared with 2% SDS containing protease inhibitors. Parasite lysates (10 μg per lane) were electrophoresed on 10% SDS-PAGE gels and transferred to a PVDF membrane, and the membranes were then incubated with mouse antibodies from immunization with mixed or fusion antigens (1:200 dilution) for 2 h. The membranes were washed three times with TBST and incubated with HRP-conjugated goat anti-mouse IgG antibodies (1:5000, Invitrogen) for 2 h at room temperature. A Pierce ECL Western Blotting Kit was used to visualize the protein on the blot (Thermo Scientific, USA). Protein loading was estimated using the anti-rHsp70 sera (Hsp70, PBANKA_0711900) produced in the laboratory.

### Indirect immunofluorescence assay (IFA)

The gametocytes were obtained from the blood of infected mice on day 4 post infection. To prevent the activation of gametocytes, the whole process was carried out on ice. The gametes were obtained by incubating the gametocyte-infected blood in a complete ookinete culture medium (RPMI 1640, 50 mg/L penicillin, 50 mg/L streptomycin, 100 mg/L neomycin, 25% [v/v] heat-inactivated fetal calf serum, 6 U/mL heparin, 100 μM xanthurenic acid, pH 8.0) for 15 min at 25 °C. Then the ookinetes were cultured for 24 h at 19 °C. The parasites were fixed with 4% paraformaldehyde and 0.0075% glutaraldehyde in 0.1 M PBS for 30 min at room temperature. After washing three times, the fixed cells were permeabilized with 0.1% Triton X-100 in PBS for 10 min and then blocked with 3% (w/v) BSA/PBS for 60 min. After blocking and another wash with PBS, parasites were probed with respective mouse antisera (1:500) at 37 °C for 1 h. In addition, parasites were co-incubated with rabbit polyclonal antisera against the female gametocyte marker P47 (1:500), the male gametocyte/gamete marker α-tubulin (1:500), the zygote and ookinete marker Pbs21 (1:500), and the gametocyte nuclear marker SET (1:500) [30]. Parasites were washed three times and then incubated with Alexa Fluor 488-conjugated goat anti-mouse IgG antibodies (1:500, Invitrogen) and Alexa Fluor 555-conjugated goat-anti-rabbit IgG antibodies (1:500, Abcam, UK) for 1 h. Then the nucleus was stained with Hoechst 33,258 (1:1000, Invitrogen) for 30 min. Secondary antibodies alone were used as negative controls. A Nikon C2 fluorescence confocal laser scanning microscope (Nikon, Japan) was used to observe fluorescence and record the images.

### Ookinete formation inhibition assay

For the in vitro assay, mice were pretreated with phenylhydrazine and injected intravenously with 5 × 10^6^ infected red blood cells (RBCs). At 3 days post injection, parasitemia was counted on Giemsa-stained blood smears. The exflagellation centers of male gametocytes and ookinetes were counted as described [25]. Briefly, 10 μL gametocyte-infected blood was mixed with immune sera that was diluted at 1:5 and 1:10 with complete ookinete culture medium in a total volume of 100 μL, and incubated at 25 °C for 15 min. One microliter of the mixture was transferred to a glass slide (Matsunami, Japan), and the exflagellation centers (10 view fields per mouse) were quantified under a phase-contrast microscope at ×400 magnification. The exflagellation centers were defined as an exflagellating male gametocyte interacting with more than four RBCs. The mixture was further incubated at 19 °C for 24 h to produce ookinetes. The number of ookinetes in 0.5 μL of culture was counted under an Olympus fluorescence microscope at ×1000 magnification after labeling with the anti-Pbs21 mAb (1:500) and Alexa-488-conjugated anti-mouse IgG antibodies (1:500) [30].

### Direct feeding assay (DFA)

The mice were immunized with the recombinant proteins (Trx-His, Pbg37, PSOP25, Pbg37+PSOP25, and Pbg37-PSOP25) as described above. Ten days after the final immunization, all mice were injected with phenylhydrazine. Three days later, 5 × 10^6^ *P. berghei*-infected RBCs were injected intravenously. Three days post infection, six mice with similar parasitemia (parasites per 100 RBCs) and gametocytemia (mature gametocytes per 10,000 RBCs) were selected for two separate experiments. Thirty adult female *An. stephensi* mosquitoes were starved for 12 h and then allowed to feed on immunized mice for 30 min. After removing the unfed mosquitoes, the engorged mosquitoes were maintained for 12 days and dissected to determine midgut infection. Midguts were stained with 0.5% Mercurochrome and oocysts were counted to assess TRA (% inhibition in mean oocyst count per mosquito) and TBA (% inhibition in the prevalence of infected mosquitoes) [31].

### Statistical analysis

Statistical analysis was performed using the SPSS software, version 21.0. The parasitemia, gametocytemia, antibody titers, exflagellation centers, and ookinetes were analyzed by analysis of variance (ANOVA). The oocyst density was analyzed by the Mann–Whitney *U* test. The prevalence of infection was analyzed by Fisher's exact test. *P* < 0.05 was considered statistically significant.

## Results

### Expression of recombinant proteins

**Fig. 1:**
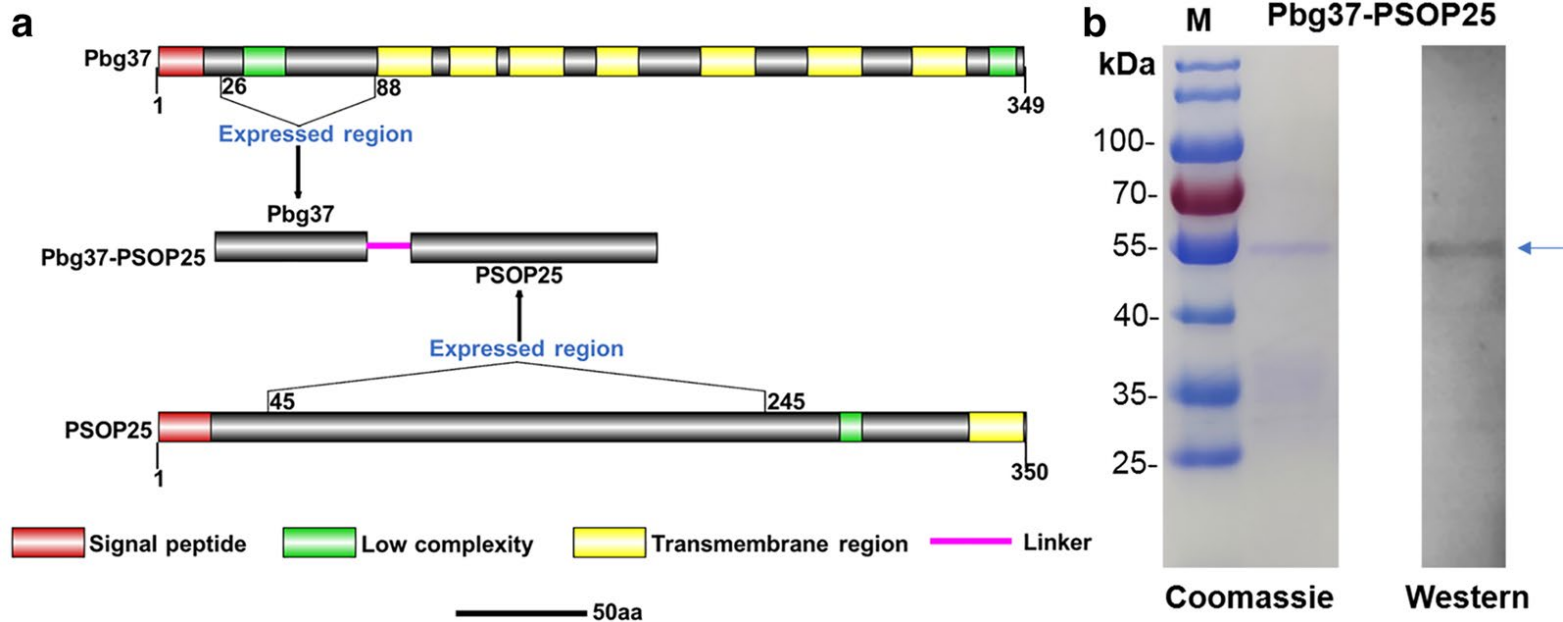
Expression and purification of the recombinant protein. **a** Diagram illustrating the expressed regions of the Pbg37, PSOP25, and their fusion Pbg37-PSOP25. The signal peptide (red box), low complexity (green box), and transmembrane region (yellow box) are highlighted. The pink line denotes the linker. **b** Purified recombinant Pbg37-PSOP25 (indicated by an arrow) was separated on a 10% SDS-PAGE gel and stained with Coomassie blue (left) and probed with anti-His mAb on a Western blot (right). M, PageRuler pre-stained protein ladder in kDa

To explore the immunogenicity of a bivalent vaccine targeting Pbg37 and PSOP25, we generated a chimeric construct containing the Lys_26_-Asn_88_ fragment of Pbg37 fused to the Met_45_-Glu_245_ fragment of PSOP25 using a flexible linker sequence (GGGGS)_3_ (Fig. 1a). Pbg37 is highly conserved among *Plasmodium* species in respective multi-sequence alignments (Additional file 2: Fig. S1A). However, the homology of PSOP25 is not high among *P. berghei*, *P. falciparum*, and *P. vivax* (Additional file 2: Fig. S1B). The expression of all recombinant proteins in *E. coli* was induced at 19 °C for 8 h to enhance protein solubility, and the expressed proteins were purified by affinity chromatography. SDS-PAGE analysis showed that the purified recombinant Pbg37-PSOP25 was homogeneous with a molecular weight of ~51 kDa (including the N-terminal Trx and His tag), consistent with its predicted size (Fig. 1b). The recombinant protein reacted with the monoclonal antibodies against the His-tag (Fig. 1b). The yield of recombinant Pbg37-PSOP25 was ~500 μg/mL, similar to that previously obtained for other recombinant proteins (Pbg37, PSOP25, and Trx).

### Immunization with recombinant proteins

**Fig. 2:**
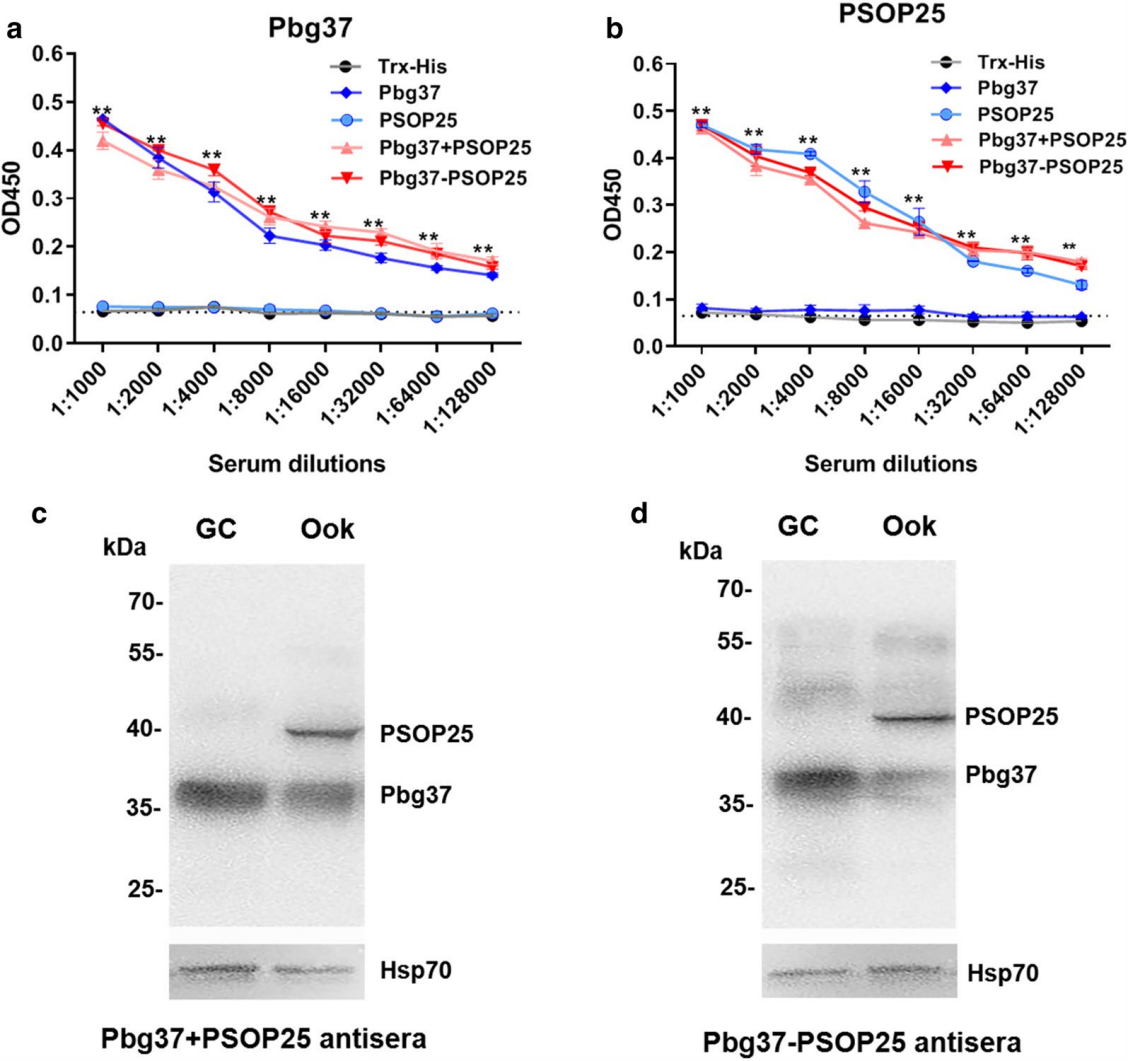
Analysis of specific antibodies by ELISA and Western blot. **a**, **b** BALB/c mice (*n* = 10) were immunized three times with Trx-His tag (immunization control) and recombinant proteins (Pbg37, PSOP25, Pbg37+PSOP25, and Pbg37-PSOP25). The equal volume sera of every mouse per group were pooled at day 10 after the final immunization for ELISA coated with the recombinant Pbg37 (**a**) and recombinant PSOP25 (**b**) polypeptides after removal of the Trx tag. Error bars indicate standard deviation. **P < 0.01 indicates a significant difference between the immunization and control groups (ANOVA). **c**, **d** The lysates of *P. berghei* gametocytes (GC) and ookinete (Ook) at 10 μg per lane were separated by 10% SDS-PAGE and probed with anti- Pbg37+PSOP25 antisera (**c**) and anti- Pbg37-PSOP25 antisera (**d**). The protein loading was estimated by the anti-rHsp70 sera

To investigate the immunogenicity of the recombinant proteins, groups of mice were immunized with the individual proteins, the two proteins together, or the chimera, and antibody titers were assessed by ELISA 10 days after the third immunization. ELISA showed that both pooled antisera and individual mouse serum of each antigen group (Pbg37, PSOP25, Pbg37+PSOP25, and Pbg37-PSOP25) yielded specific antibodies against Pbg37 and/or PSOP25 (Fig. 2a, Fig. S2). As expected, immunization with individual antigens yielded only the antibodies specific for the respective antigens used for immunization. The antibody titers against Pbg37 induced by recombinant Pbg37 alone or mixed/fused with PSOP25 were similar to each other (Fig. 2a, Additional file 2: Fig. S2B–D). Similarly, the antibody titers against PSOP25 induced by recombinant PSOP25 alone, mixed, or fused with Pbg37, were also comparable (Fig. 2b, Fig. S2F–H), indicating no immune interference between the two antigens.

### Reactivity of the antisera with the native proteins of the parasite

Pbg37 was expressed primarily in gametocytes and gametes, and to a lesser extent, in zygotes and ookinetes, whereas PSOP25 was expressed on the outer surface of the ookinetes [25, 26]. To verify that the antisera produced against the antigen mixture or chimera reacted with the native proteins in the parasites, Western blot analysis was performed using lysates from gametocytes and ookinetes. Both antibodies against the mixture and chimera of Pbg37 and PSOP25 detected a single band at ~37 kDa of the gametocyte lysates, whereas in the ookinete lysates, the antibodies identified a more prominent protein band at ~40 kDa in addition to the ~37 kDa band (Fig. 2c, d). The 37 kDa and 40 kDa bands correspond to the sizes of the Pbg37 and PSOP25 proteins, respectively, detected earlier in these parasite stages [25, 26].

**Fig. 3:**
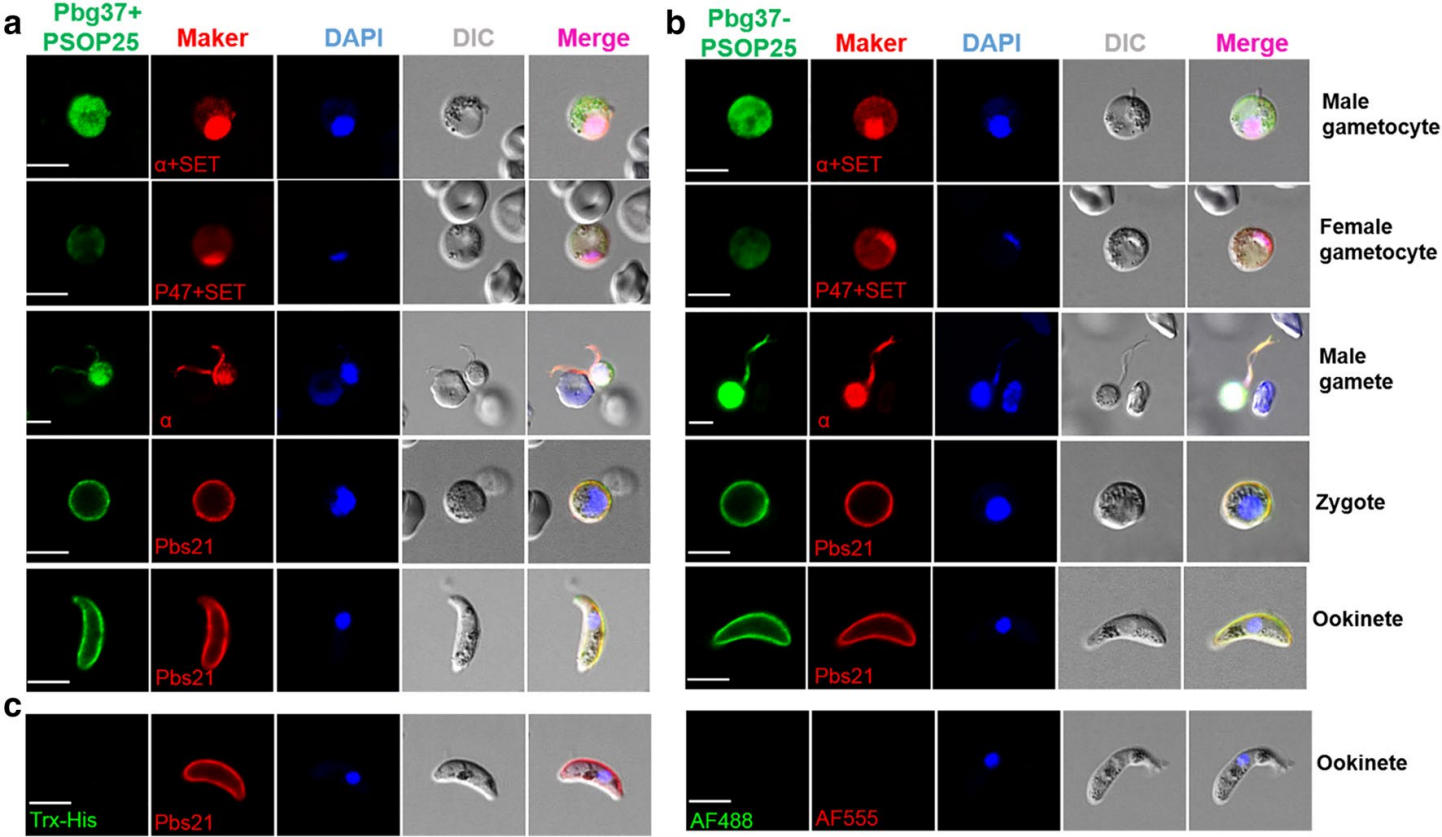
Indirect immunofluorescence analysis using the bivalent immune sera. Parasites of different developmental stages were fixed and permeabilized with 0.1% Triton X-100. They were stained with antisera against Pbg37+PSOP25 (**a**) and Pbg37-PSOP25 (**b**) (1:200) as the primary antibodies (green). The parasites were also co-labeled with antibodies against the markers for different stages (red), including α-tubulin (α) for male gametocytes/gametes, P47 for female gametocytes, Pbs21 for zygotes and ookinetes, and SET for the nucleus of gametocytes. Alexa Fluor 488-conjugated goat anti-mouse IgG antibodies and Alexa Fluor 555-conjugated goat anti-rabbit IgG antibodies were used as the secondary antibodies. **c** Negative controls showing the ookinetes labeled with the Trx-His antisera or with the secondary antibodies only. The nucleus was stained with Hoechst-33258 (blue). Images were obtained under the same conditions at a magnification of ×1000. *DIC* differential interference contrast microscopy. Scale bar = 5 μm

To further characterize the specificity of the antibodies, IFA was performed with different sexual stages of the parasite. Both antisera against the antigen mixture and fused antigen reacted with the gametocytes, gametes, zygotes, and ookinetes (Fig. 3a, b). It is noteworthy that the fluorescence intensity was higher in male gametocytes/gametes than female gametocytes/gametes. In the exflagellating male gametes, the fluorescence was mostly associated with the residual body, although the flagella were also labeled. In zygotes and ookinetes, strong fluorescence was associated with the plasma membranes (Fig. 3a, b). Negative controls performed with antibodies against Trx-His or with only the secondary antibodies did not react with these sexual stages (Fig. 3c). Collectively, antisera against the mixed or fused antigens from the pre- and post-fertilization stages were able to recognize the respective proteins expressed during the entire gametocyte-ookinete development.

### Transmission-blocking activities

**Fig. 4:**
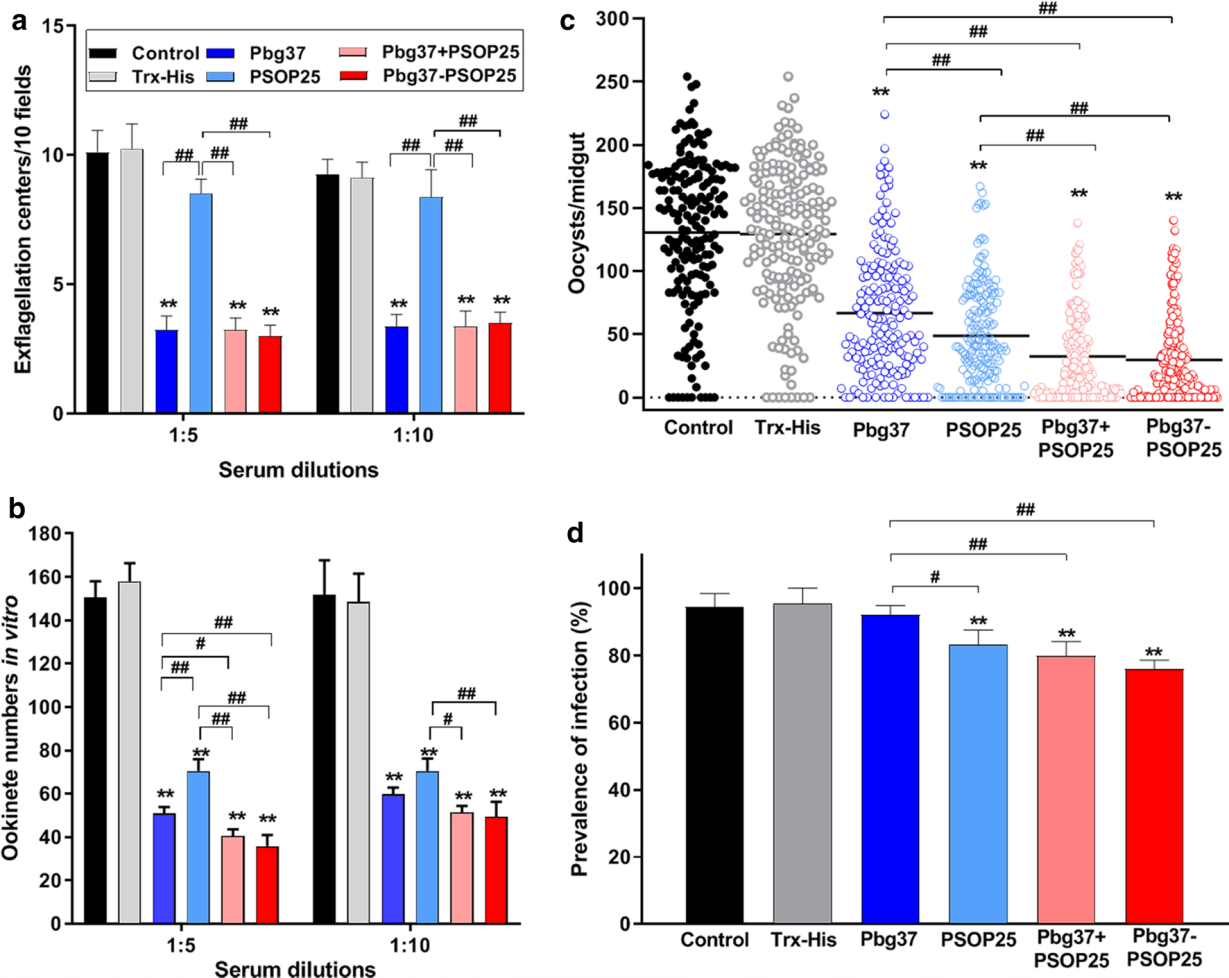
Transmission-blocking activities of the antisera assessed in vitro and in vivo. **a** Inhibition of exflagellation. *P. berghei*-infected blood collected at 3 days post infection was incubated with the respective control and immune sera at dilutions of 1:5 and 1:10. Exflagellation centers in 10 microscopic fields were counted after 15 min. **b**) Inhibition of ookinete formation. Under the same culture conditions, ookinetes formed within 24 h were stained with Pbs21 mAb and counted. Data for exflagellation and ookinetes were representative of three independent experiments. **c** Midgut oocysts were counted from individual mosquitoes infected with parasites 12 days after the blood meal. The results were collected from three mice in each immunization group and in two separate experiments (*n* = 180). Data points represent oocyst numbers in individual mosquitoes. Horizontal bars indicate the mean number of oocysts per midgut. **d** Mosquito infection prevalence was calculated at 12 days after the blood meal. Data points represent the prevalence of infection in mosquitoes from three mice per group and in two separate experiments. Error bars indicate mean ± SD. *P < 0.05 and **P < 0.01 represent the significant difference between the respective immunization group and the Trx-His control group. ^#^*P* < 0.05 and ^##^*P* < 0.01 represent significant difference between two immunization groups

To determine whether antisera against mixed or fused antigens could improve TBA, these antisera were first assessed using in vitro exflagellation and ookinete formation inhibition assays. Consistent with previous studies, the Trx-His immunization and non-immunized control showed very similar results in both exflagellation and ookinete formation assays (Fig. 4a, b). When *P. berghei* gametocytes were incubated with the control and immune sera, exflagellation of male gametocytes was inhibited by the antisera against Pbg37, Pbg37+PSOP25, and Pbg37-PSOP25 by 68%, 68%, and 71% at 1:5 dilution, and by 63%, 63%, and 62% at 1:10 dilution, respectively, as compared to the Trx-His immunization group (Fig. 4a). In contrast, antisera against PSOP25 showed no significant impacts on exflagellation. The ookinete numbers were counted 24 h after incubation, and all antisera against the Pbg37, PSOP25, their mixture, and the chimera protein showed significant inhibition of ookinete formation (Fig. 4b). The ookinete numbers of the antisera against Pbg37+PSOP25 and Pbg37-PSOP25 at 1:5 dilution were 40.5 and 35.8, corresponding to a reduction of ookinete formation by 74% and 77%, respectively, compared to the Trx-His group. In comparison, antisera against single antigens Pbg37 and PSOP25 reduced ookinete formation by 68% and 55%, respectively. A similar trend of reduction of in vitro ookinete formation was observed when these antisera were used at 1:10 dilution. The ookinete numbers with the antisera against Pbg37+PSOP25 (51.5) and Pbg37-PSOP25 (49.5) were significantly larger than the number at 1:5 dilution (Fig. 4b). The result showed that the sera against mixed and fused antigens produced stronger inhibition effects on ookinete formation than the antisera against individual antigens, and effects of the immune sera against the mixed and fused antigens on ookinete formation were concentration-dependent.

To further evaluate the TBA of the antibodies in vivo, mice immunized with the respective recombinant antigens were infected with *P. berghei* and used for direct mosquito feeding. Since within each group the antibody titers against the respective antigens were similar among the ten mice, the mice with a similar parasitemia and gametocytemia were selected for DFA (Fig S3). Given that the oocyst density and infection prevalence data were similar for each immunization group in the two experiments, data from all six mice were combined and presented in Fig. 4c, d and Table 1, while the data from individual mice are shown in Additional file 2: Supplemental Fig. S4 and Additional file 3: Table S2. As expected, the Trx-His immunization control showed similar oocyst densities and infection prevalence as the naïve control (Fig. 4c, d). Compared with the Trx-His immunization control, midgut oocyst densities in all the immunization groups were significantly reduced by 48.4–77.0% (*P* < 0.01, Mann–Whitney *U* test; Fig. 4c, Table 1). Except for the Pbg37 group, the mosquito infection prevalence in all immunization groups was also significantly reduced by 12.8–19.5% (*P* < 0.01, Fisher’s exact test, Fig. 4d, Table 1). Furthermore, there were significant reductions in oocyst density in the mixed-antigen (74.9%) and fused-antigen (77.0%) immunization groups compared with the Pbg37 (48.4%) or the PSOP25 (62.3%) immunization groups (*P* < 0.01, Mann–Whitney *U* test; Fig. 4c, Table 1). With regard to the infection prevalence (TBA), the PSOP25, Pbg37+PSOP25 and Pbg37-PSOP25 groups also exhibited significant decreases relative to the Pbg37 immunization group (*P* < 0.05, Fisher’s exact test; Fig. 4d, Table 1). Collectively, these mosquito feeding assays demonstrated that the two sexual-stage antigens Pbg37 and PSOP25, when mixed or fused for immunization, produced significantly higher TRA than when they were used individually. In most mice, immunization with the fusion proteins produced slightly lower infection prevalence and intensity than when they were mixed for immunization, although the results were not statistically significant (Additional file 2: Fig. S4, Additional file 3: Table S2).

**Table 1:** Evaluation of the transmission-blocking activity of different immunization groups in mosquito feeding assays

Mouse	Immunization group	Oocyst density mean (range) (*n* = 180)	TRA^a^	Prevalence of infection mean (*n* = 180)	TBA^b^
Mouse 1–6	Control	130.6 (0–254)		94.4% (170/180)	
	Trx-His	129.4 (0–254)		95.6% (172/180)	
	Pbg37	66.8 (0–224)	48.4%	92.2% (166/180)	3.4%
	PSOP25	48.7 (0–167)	62.3%	82.8% (149/180)	12.8%
	Pbg37+PSOP25	32.4 (0–138)	74.9%	80.0% (144/180)	15.6%
	Pbg37-PSOP25	29.7 (0–140)	77.0%	76.1% (137/180)	19.5%

^a^TRA was calculated as (mean oocyst density_Trx-His_ – mean oocyst density _Pbg37/PSOP25/Pbg37+PSOP25/Pbg37-PSOP25_) / mean oocyst density_Trx-His_ × 100%

^b^ TBA was calculated as % prevalence_Trx-His_ – % prevalence_Pbg37/PSOP25/Pbg37+PSOP25/Pbg37-PSOP25_

## Discussion

TBVs combining two different antigens may be more effective than single antigen-based TBV, presumably by increasing the TBA at low antibody titers and by increasing the proportion of the vaccinated population with effective levels of TBA [32]. The effects of such bivalent vaccines would be further enhanced if there was a synergy between the two antigens, as suggested for Pfs25 and Pfs28 [22]. However, combination vaccines also carry the risk of immunological interference between the antigens, which could lead to reduced immune responses to one or more components. In this study, two sexual-stage antigens Pbg37 and PSOP25 were evaluated for a bivalent TBV using the rodent malaria model. Under the immunization conditions used, there was no obvious immune interference between the two antigens. Moreover, these two antigens, either as mixed proteins or as a fusion protein, elicited a significantly higher TRA than these antigens used separately, suggesting a synergy between them.

The Pbg37 and PSOP25 were selected for the evaluation of a bivalent TBV because these two proteins are expressed predominantly in different sexual stages, and their small fragments (62 and 200 aa) can elicit strong antibody responses with excellent TBA [25, 26]. These two protein fragments have been expressed successfully in *E. coli* with excellent solubility and yield as well as correct folding as suggested by the TBA induced after immunization in mice. In designing fusion proteins, different strategies have been used in the selection of the linkers. For example, Menon et al. used viral vectors to generate a dual-antigen TBV of Pfs25 and Pfs230C with either a GP linker to produce a fusion protein or with the picornavirus 2A linker sequence for self-cleavage of the recombinant proteins within the linker [18]. In the design of the Pbg37-PSOP25 fusion protein, there was a linker (GGGGS)_3_ composed of four small, nonpolar (Gly) amino acids and a polar (Ser) amino acid to provide flexibility and mobility of the functional domains. The Ser residue would keep the linker stable by forming hydrogen bonds in aqueous solutions, thus reducing negative interactions between the linker and the protein moieties. The copy number was set at three to attain a proper separation of the two parts [33]. The resulting Pbg37-PSOP25 fusion protein was relatively small in size (~265 aa) and maintained excellent solubility, yield, and conformation when expressed in *E. coli*, validating the chimeric design.

Immunological interference effects should be assessed when generating a dual-antigen vaccine. The interference could change both the quantity and quality of the induced antibodies. The combination of different virus-vectored malaria vaccines reportedly results in substantial immune interference [19, 34]. The combination of RTS,S/AS01 with a viral vector PEV (ChAd63/MVA ME-TRAP) failed to improve the vaccine efficacy when compared to the two vaccines administered individually [35]. For a bivalent TBV design, the combination of Pfs25 and Pfs230C1 did not enhance the antibody response when compared to single antigen immunization [23]. In this study, specific antibody responses against the Pbg37 and PSOP25 fragments were tested, and the results demonstrated that these two protein fragments did not interfere with each other in inducing functional antibodies. ELISA showed that these two sexual-stage antigens administered in mice, either as mixed proteins or a fusion protein, elicited similar antibody levels against Pbg37 or PSOP25 as these proteins when used individually. In vitro studies demonstrated that the antibodies from the bivalent vaccines significantly reduced exflagellation (due to the activity of the anti-Pfg37 antibodies) and ookinete development (mostly due to the activity of the anti-PSOP25 antibodies), indicating the functional activity of the antibodies produced against the mixed or chimeric proteins. With extensive washes during the purification step and dialysis of the purified proteins, endotoxin was not quantified in the samples. Future studies should quantify the endotoxin to exclude potential interference with the immunization and toxicity in mice.

By selecting two antigens targeting different sexual stages of the parasite, this study expanded the portion of the sexual development during which the transmission-blocking antibodies function, and showed that the dual-antigen vaccines elicited antigen-specific antibodies against both the native Pbg37 that is mainly expressed in gametocytes/gametes, and PSOP25 that is expressed on ookinetes. Antibodies against Pbg37 mostly inhibited exflagellation and fertilization with minor effects on ookinete development, whereas those against PSOP25 mainly hindered ookinete development (Fig. 4a, b). In comparison, the bivalent vaccine with Pfs25 and Pfs230 did not show improvement in TRA regardless of the antibody concentrations in mice [23]. Antibodies produced in rabbits did not show enhanced TRA in the standard membrane feeding assay. The observed significant improvement of TBA from the bivalent vaccine Pbg37 and PSOP25 compared with these antigens used alone is very likely due to the synergistic effects of the antibodies against the two antigens to cover the entire gametocyte-ookinete development. Moreover, slightly better TBA with the chimeric protein was observed than the protein mixture. While the reasons for this minor difference were not investigated, it is speculated that the chimeric construct might better preserve the conformation of the two protein fragments, resulting in antibodies with increased avidity to the native proteins of the parasites [18].

## Conclusion

This study evaluated two recently identified TBV candidates expressed primarily in different sexual stages for a bivalent TBV design. The results showed no immunological interference between the two antigens. The superior TBA with the antibodies produced with the two bivalent vaccines (mixed proteins or a fusion protein) as compared to that of the antibodies against individual antigens is probably due to their action at both the pre- and post-fertilization stages. These promising results provide the basis for future evaluation of these antigens in human malaria parasites.

## Supplementary Information

**Additional file** **1: Table S1.** Integration-specific primers for protein expression vector.

**Additional file 2: Figure S1.** Sequence analysis of Pbg37 and PSOP25. **Figure S2.** Antibody titers in individual mouse. **Figure S3.** The parasitemia and gametocytemia of individual immunized mice used in the two DFA experiments. **Figure S4.** Transmission-reducing potential of antisera in vivo.

**Additional file 3: Table S2.** Evaluation of transmission-blocking effect of different immunization groups in mosquito feeding assays.

## Data Availability

The data supporting the conclusions of this article are included within the article.
