# Supplementary material for: Evaluation of two sexual-stage antigens as bivalent transmission-blocking vaccines in rodent malaria
Source: Parasit Vectors. 2021 May 7;14:241. doi: 10.1186/s13071-021-04743-0 (PMC8103607; doi:10.1186/s13071-021-04743-0)
Supplement: Supplementary file 2 — Additional file 2: Figure S1. Sequence analysis of Pbg37 and PSOP25. Figure S2. Antibody titers in individual mouse. Figure S3. The parasitemia and gametocytemia of individual immunized mice used in the two DFA experiments. Figure S4. Transmission-reducing potential of antisera in vivo. [file 13071_2021_4743_MOESM2_ESM.pdf]

Fig S1

A.

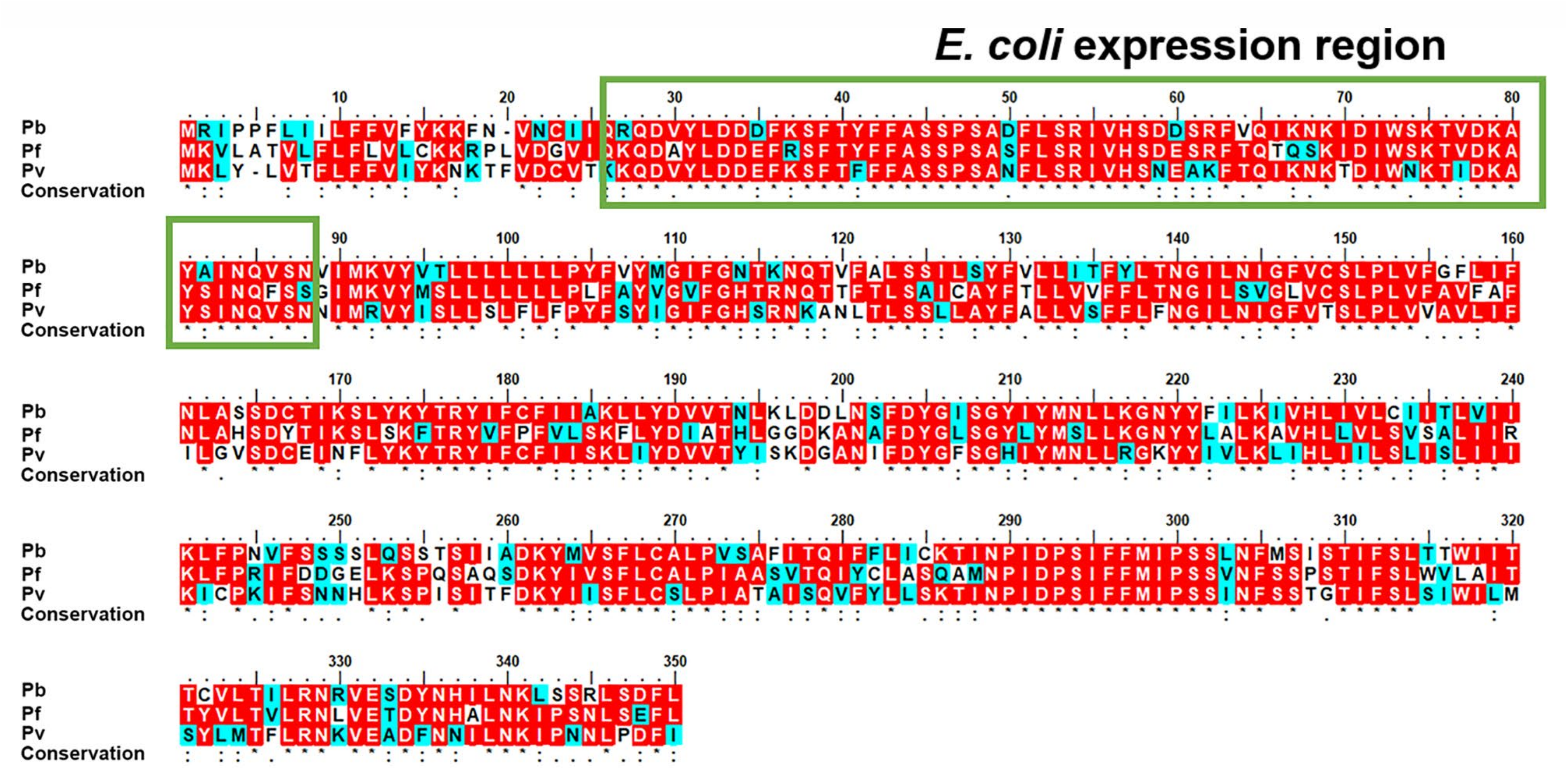

Fig S1

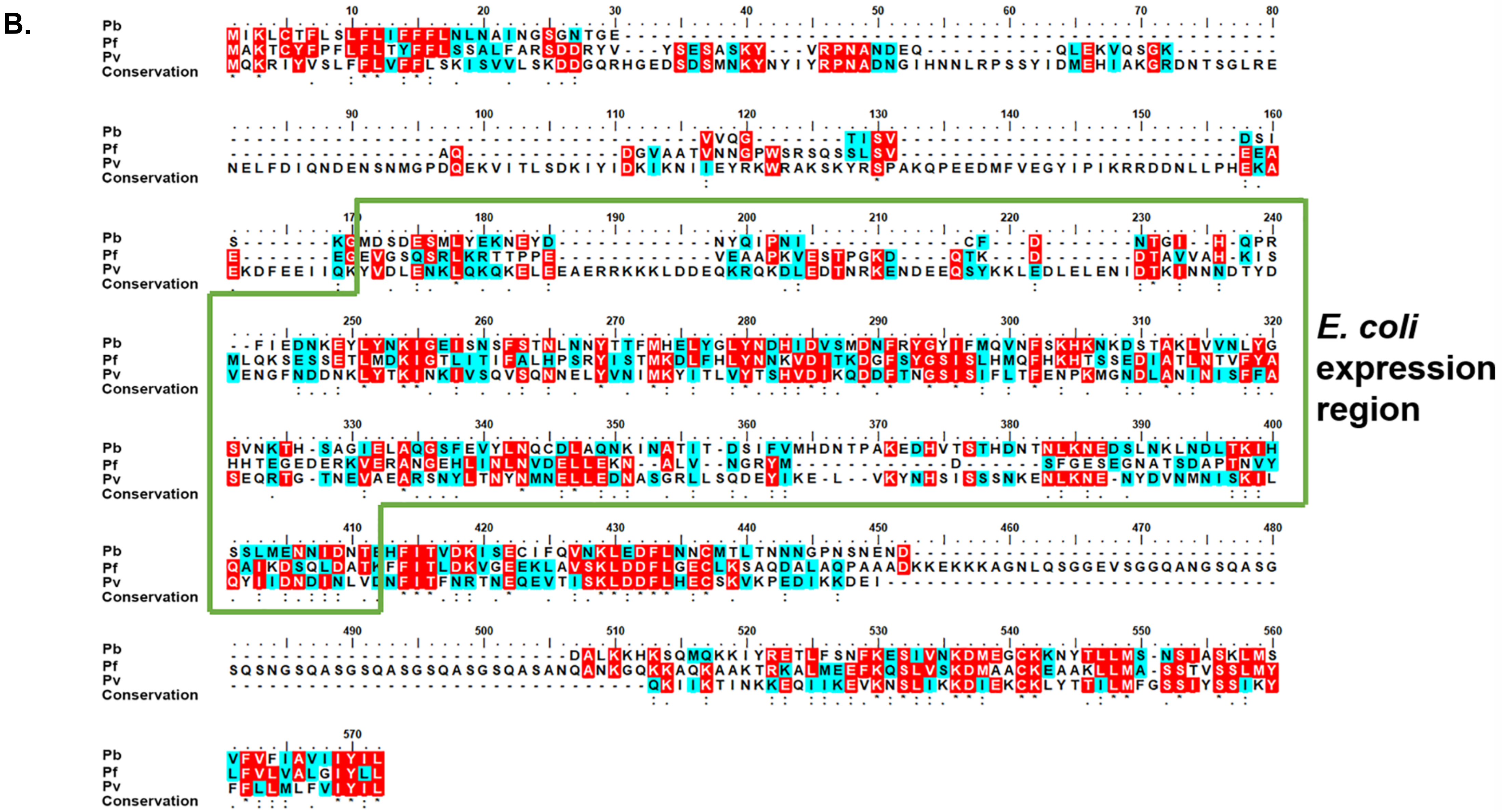

Fig. S1. Sequence analysis of Pbg37 and PSOP25. Alignment of Pbg37 **(A)** and PSOP25 **(B)** proteins in *P. berghei* (*Pb*), *P. falciparum* (*Pf*) and *P. vivax* (*Pv*) . Amino acids conserved across three species are marked in red for identical and blue for similar residues. The fragments (Pbg37: 26-88 aa, PSOP25: 45-245 aa) expressed in *E. coli* were indicated.

Fig S2

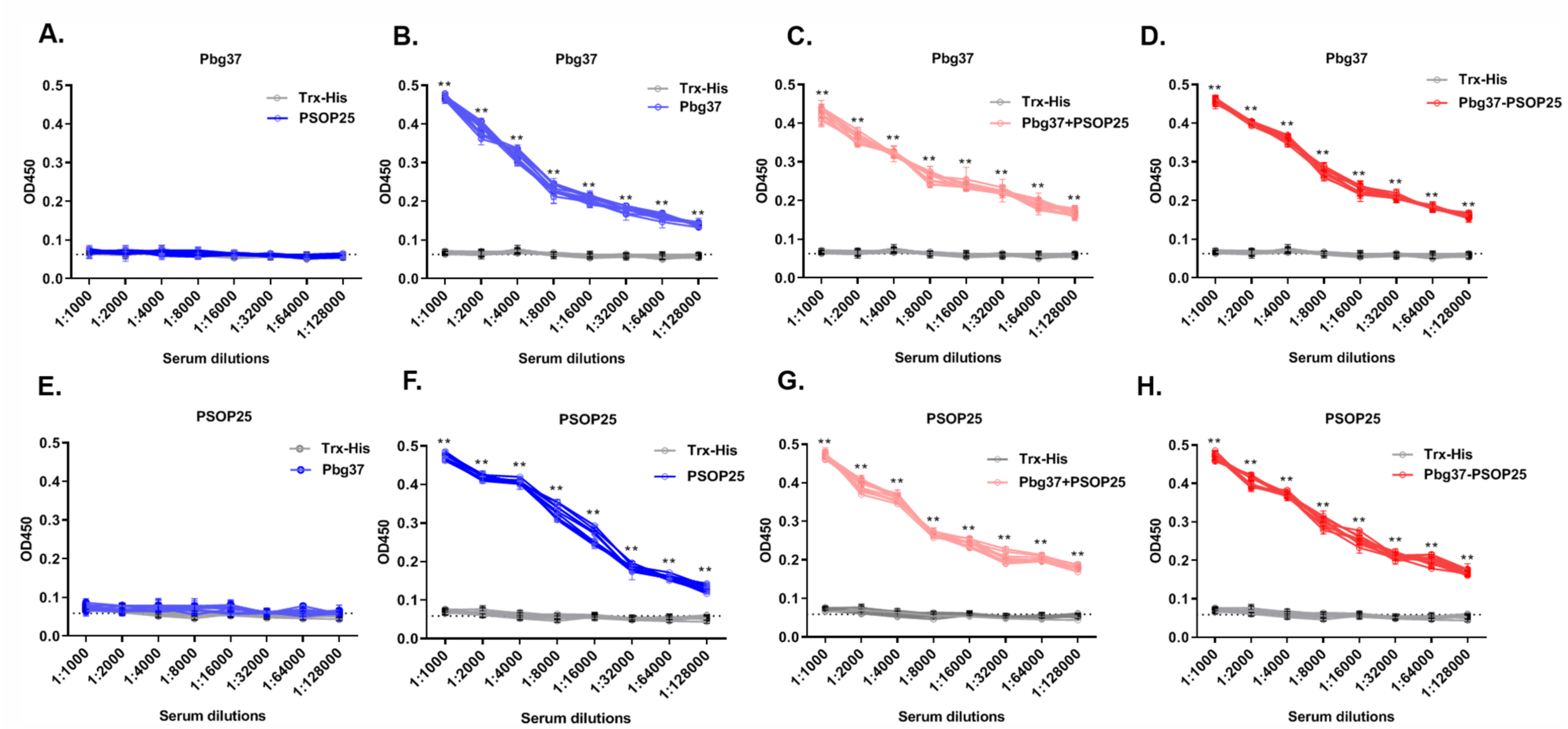

Fig. S2. Antibody titers in individual mouse. Antibody titers against Pbg37 or PSOP25 in individual mice were tested by ELISA. The 96-well plates were coated with purified Pbg37 **(A-D)** or PSOP25 **(E-H)** recombinant proteins after the removal of the Trx tag. The serum of each mouse in the PSOP25 **(A, F)** , Pbg37 **(B, E)** , Pbg37+PSOP25 **(C, G)**, Pbg37-PSOP25 **(D, H)** groups collected at day 10 after the final immunization was evaluated by ELISA separately. ***P* < 0.01 represents the difference between the recombinant protein immunization groups with Trx-His control group.

Fig S3

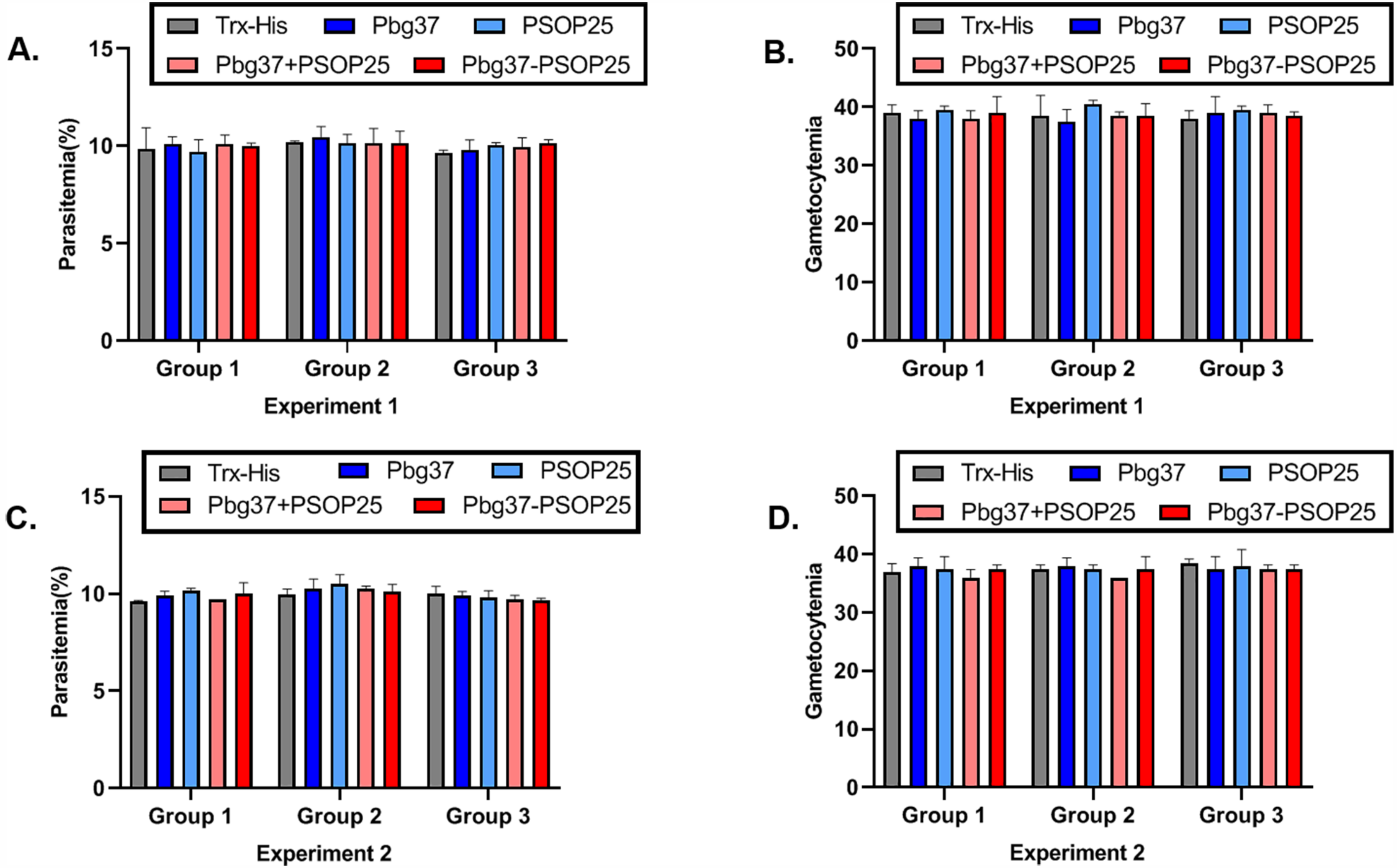

Fig. S3. The parasitemias and gametocytemias of individual immunized mice used in the two DFA experiments. BALB/c mice immunized with respective recombinant proteins were infected with *P. berghei* parasites. The parasitemias and gametocytemias of individual mice were measured in Experiment 1 **(A, B)** and Experiment 2 **(C, D)**.

Fig S4

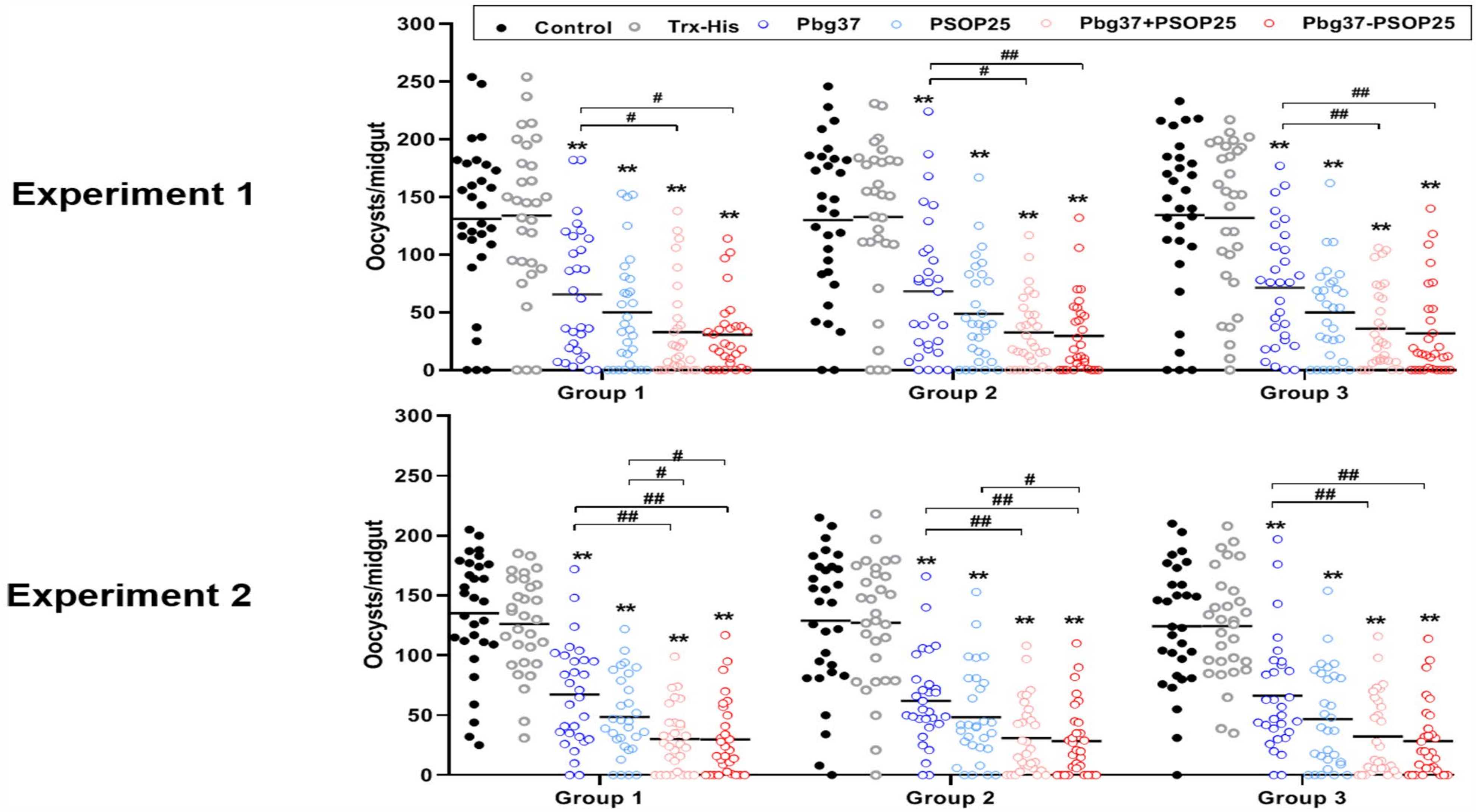

Fig. S4. Transmission-reducing potential of antisera in vivo. BALB/c mice immunized with respective recombinant proteins was infected with *P. berghei* parasites 10 days after the third immunization. Mosquitoes were allowed to feed on the infected mice after 3 days. Data points represent oocyst numbers in individual mosquitoes 12 days post blood meal. Horizontal bars indicate the mean number of oocysts per midgut from mosquitoes fed on each mouse (n = 30). Group 1-3 represent three different biological replicates in two separate experiments, respectively. **P* < 0.05 and ***P* < 0.01 represents the difference between the recombinant protein immunization groups with Trx-His control group. #*P* < 0.05 and ##*P* < 0.01 represents the difference among the different recombinant protein immunization groups (Mann-Whitney *U* test).
